# A Nucleotide Signature for the Identification of Angelicae Sinensis Radix (Danggui) and Its Products

**DOI:** 10.1038/srep34940

**Published:** 2016-10-07

**Authors:** Xiaoyue Wang, Yang Liu, Lili Wang, Jianping Han, Shilin Chen

**Affiliations:** 1 Institute of Medicinal Plant Development, Chinese Academy of Medicinal Science &Peking Union Medicinal College, Beijing 100193, P. R. China; 2 Institute of Chinese Materia Medica, China Academy of Chinese Medical Sciences, Beijing 100700, P. R. China

## Abstract

It is very difficult to identify Angelicae sinensis radix (Danggui) when it is processed into Chinese patent medicines. The proposed internal transcribed spacer 2 (ITS2) is not sufficient to resolve heavily processed materials. Therefore, a short barcode for the identification of processed materials is urgently needed. In this study, 265 samples of Angelicae sinensis radix and adulterants were collected. The ITS2 region was sequenced, and based on one single nucleotide polymorphism(SNP) site unique to *Angelica sinensis*, a nucleotide signature consisting of 37-bp (5′-aatccgcgtc atcttagtga gctcaaggac ccttagg-3′) was developed. It is highly conserved and specific within *Angelica sinensis* while divergent among other species. Then, we designed primers (DG01F/DG01R) to amplify the nucleotide signature region from processed materials. 15 samples procured online were analysed. By seeking the signature, we found that 7 of them were counterfeits. 28 batches of Chinese patent medicines containing Danggui were amplified. 19 of them were found to contain the signature, and adulterants such as *Ligusticum sinense*, *Notopterygium incisum*, *Angelica decursiva* and *Angelica gigas* were detected in other batches. Thus, this nucleotide signature, with only 37-bp, will broaden the application of DNA barcoding to identify the components in decoctions, Chinese patent medicines and other products with degraded DNA.

Angelicae sinensis radix (Danggui) derived from *Angelica sinensis* (Oliv.) Diels, is a well-known Pharmacopoeia-recorded material medica in China^1^. It has been called “female ginseng” due to its superior efficacy in treating gynaecological conditions^2^. Used primarily to treat anaemia^3^, its medicinal value has been demonstrated by numerous clinical trials, pre-clinical studies and traditional or modern experiments^4^,^5^,^6^,^7^. Angelicae sinensis radix is a commonly used herbal medicine that is found in approximately 100 Chinese patent medicines recorded in the Chinese Pharmacopoeia^1^. It lends itself to wide application, as expressed in the adage “Ten prescriptions nine Danggui”. Due to increasing demand, many adulterants have emerged on the market, including Angelicae pubescentis radix (Duhuo), derived from *Angelica biserrata,* whose synonym is *Angelica pubescens* f. *biserrata*; Ligustici rhizoma et radix (Gaoben), derived from *Ligusticum sinense* or *Ligusticum jeholense*; Peucedani decursivi radix (Zihuaqianhu), derived from *Angelica decursiva*, whose synonym is *Peucedanum decursivum*; Notopterygii rhizoma et radix (Qianghuo), derived from *Notopterygium incisum* or *Notopterygium franchetii*; the root of *Levisticum officinale*, *A. gigas*, *A. magaphylla*, *A. valida*, *A. acutiloba* also can be adulterants with Angelicae sinensis radix. Among these, *A. gigas* is used as “Danggui” in Korea and Yanbian, Jilin Province; *A. acutiloba* is also a statutory “Danggui” species recorded in the Japanese Pharmacopoeia. Because it has morphological characters that are similar to those of its adulterants, taxonomic identification based on morphology is very difficult, especially after it has been processed into Chinese patent medicines. Previous studies have reported that ferulic acid can be used as a chemical marker to detect Angelicae sinensis radix in Chinese herbal decoctions via HPLC^8^, but ferulic acid has been commonly found in many related plants^9^,^10^,^11^. Microscopy is another primary method of identification of Chinese patent medicines^12^; however, sometimes microscopic characteristics lead to ambiguous identifications. Moreover, microscopic characteristics are undetectable when herbs are ground into ultrafine powders with a diameter less than 10 microns. Therefore, a rapid method for the precise analysis of Angelicae sinensis radix products is highly desired.

DNA barcoding technology uses a standard gene fragment for rapid identification of unknown specimens to species level. Its accuracy and repeatability have made it successful in species identification^13^,^14^. ITS/ITS2 sequences have been extensively tested and evaluated as candidate DNA barcodes in the plant kingdom^15^. Our previous study also showed that ITS2 can be used as a mini-barcode to effectively identify species in a wide variety of specimens and medicinal materials, based on analyses of 12,861 ITS and ITS2 sequences^16^, and ITS2 has been widely used for herb identification. For instance, Xin *et al*. used ITS2 to distinguish commercial *Rhodiola* products successfully and found that 60% contained adulterants^17^; Zhao *et al*. identified Acanthopanacis cortex and its adulterants with ITS2^18^. He *et al*. designed specific ITS primers to identify *A. sinensis* and its adulterants^19^. However, ITS is not able to distinguish *A. sinensis* from Chinese patent medicines or decoctions. Meusnier *et al*. proposed a “mini-barcode” sequence to overcome the amplification problem of degraded DNA^20^. They found that short lengths of 150-bp and 100-bp in the CO1 region could achieve 95% and 90% successful identification, respectively. Dubey used a 175-bp region to distinguish 11 endangered snake species of India^21^. Shaw *et al*. successfully obtained an 88-bp fragment from TCM decoction, but the amplification of the longer fragment was unsuccessful^22^. Shokralla *et al*. designed six mini-barcodes (127~314-bp) to detect processed fish products. While the 650-bp length (CO1) could distinguish only 20.5% of the species tested, mini-barcoding achieved 88.6% successful identification, indicating that shorter fragment lengths are more likely to be amplified from degraded DNA^23^. The phrase “nucleotide signature” refers to one or more nucleotides unique to one species, and a nucleotide signature has been used in the identification of *Aglaia stellatopilosa*^24^. Liu *et al*. successfully developed a 23-bp signature for identification of ginseng products and found some adulterants in Chinese patent medicines^25^. These studies have inspired us to develop a short nucleotide signature for Angelicae sinensis radix within the ITS2 region.

In this study, we aimed to develop a short gene identifier for the identification of Angelicae sinensis radix and its Chinese patent medicines. A 37-bp (5′-aatccgcgtc atcttagtga gctcaaggac ccttagg-3′) nucleotide signature was found. This exclusive nucleotide signature of Angelicae sinensis radix is completely conserved within the *A. sinensis* species. The method was also used for analysis of Chinese patent medicines containing Angelicae sinensis radix.

## Results

### Development of Nucleotide Signature for *A. sinensis*

A total of 265 ITS2 sequences of *A. sinensis* and closely related species from experimental materials and our previous study were aligned, and the aligned length was 454-bp long. The results showed that 126 sequences of the *A. sinensis* species were highly conserved. Simultaneously, a SNP at position 193 was found to be unique to *A. sinensis*. Based on this SNP site, we developed a 37-bp nucleotide signature (5′-aatccgcgtc atcttagtga gctcaaggac ccttagg-3′) exclusively specific for *A. sinensis*.

**Figure 1:**
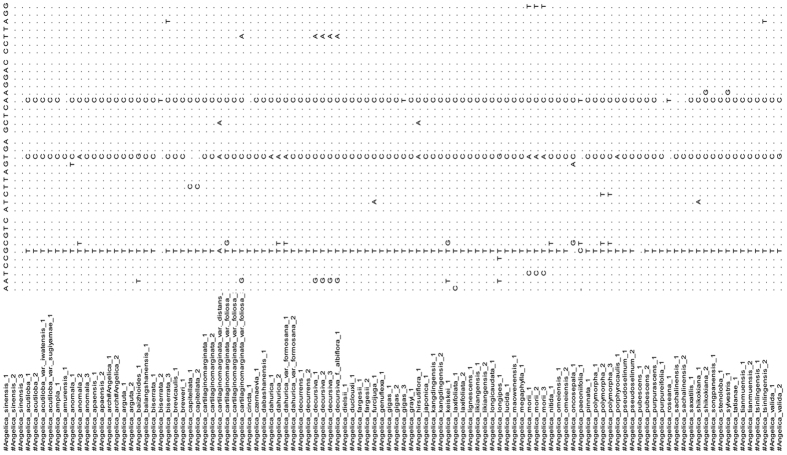
The alignment of the 37-nucleotide conserved region.

A total of 105 haplotypes representing the 429 ITS2 sequences of the closely related *Angelica* species from GenBank were annotated to this 37-bp nucleotide signature region and were aligned using MEGA 5.0 (Fig. 1). In this highly conserved region of *A. sinensis*, other species had 1 to 5 divergent nucleotides. Therefore, this region was defined as the nucleotide signature of *A. sinensis*. This regions of all the individuals within *A. sinensis* species were completely conserved. No variable sites were found among them.

### Verification of the nucleotide signature in decoction and extract powder

**Figure 2:**
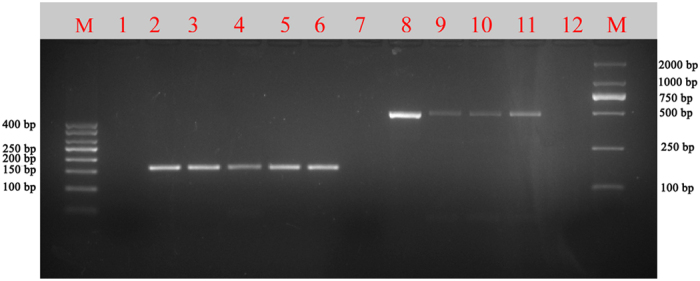
Two primer pairs amplify different sizes of sequences from decoctions and extract powder. Note: Except for the first and last lanes (marker), the remaining 12 lanes are divided into two groups for two primer pairs. Each primer pair occupies six lanes. Lanes from left to right are DG01F/DG01R and ITS2F/3R. The first lane in each primer pair (lane 1 and 7) is a negative control. The second lane (lane 2 and 8) is a positive control without boiling. The third to fifth lanes (lane 3, 4, 5, 9, 10 and 11) are PCR products from decoctions with boiling times of 120 min, 180 min and 240 min, respectively. The sixth lane (lane 6 and 12) is a PCR product from extract powder.

We used the decoction and Danggui extract powder to verify whether the nucleotide signature method functions with processed materials. We purchased 15 Angelicae sinensis radix products online including 9 batches of “Danggui powder”, 1 batch of “Danggui extract powder”, 2 batches of “Danggui whole roots” and 3 batches of “Danggui slices”. We also boiled “Danggui powder” to prepare the decoctions. The complete ITS2 region (approximately 450-bp) was amplified and sequenced using the universal primer pair ITS2F/3R. In addition to “Danggui extract powder” and the decoctions, 13 other products were successfully amplified and sequenced. We designed a specific primer pair to amplify the signature from the degraded DNA. The results showed that the shorter barcode can still be amplified and that the length of amplification is 167-bp (Fig. 2). The PCR products were successfully sequenced and clean trace files were generated. Via analysis of nucleotide signature, seven powders were found to be counterfeit, which were identified as Angelicae pubescentis Radix (Duhuo) (Table 1).

### Validation of the nucleotide signature and specific primers for identification of the decoctions and Chinese patent medicines

There are many types of Chinese patent medicines containing Angelicae sinensis radix. Most Chinese patent medicines contain different species, and we selected Huoxue Zhitong Capsule for the preliminary experiment. Huoxue Zhitong Capsule contains Angelicae sinensis radix and 5 other ingredients as follows: Notoginseng Radix et Rhizoma (Sanqi), Olibanum (Ruxiang), Borneolum Syntheticum (Bingpian), Eupolyphaga Steleophaga (Tubiechong) and Pyritum (Zirantong). We tested the availability of the specific designed primer pair DG01F/DG01R to amplify the nucleotide signature region of Angelicae sinensis radix from this Chinese patent medicine. The results showed that the targeted nucleotide signature of Angelicae sinensis radix was successfully amplified and sequenced from the 2 batches of Huoxue Zhitong Capsules.

**Figure 3:**
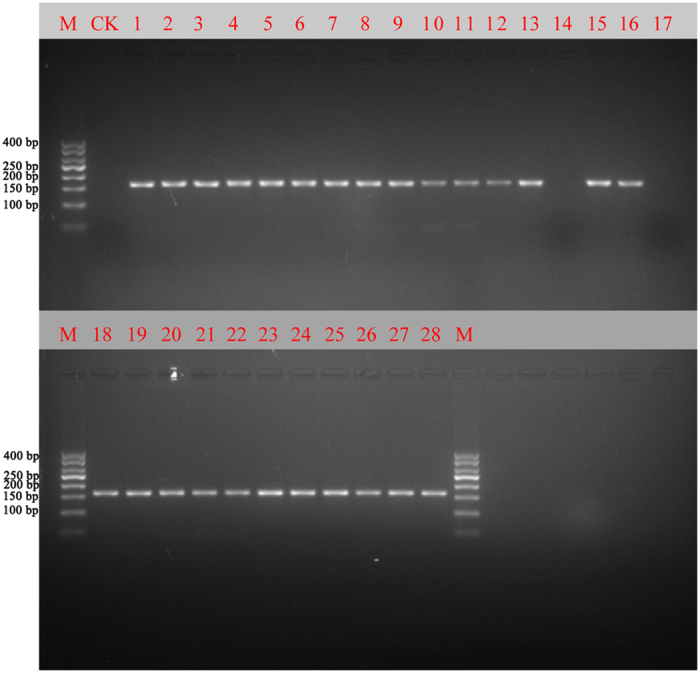
PCR amplifications of the 28 Chinese patent medicines with DG01F/DG01R. Note: Except for the lanes marked M (marker) and CK (positive control), the remaining 28 lanes contain PCR products from the 28 Chinese patent medicines. The order is the same as in Table 2.

The other 26 batches of Chinese patent medicines containing Angelicae sinensis radix were also tested, including Wuji Baifeng Pills, Deida Pills, Renshen Jianpi Pills. All the Chinese patent medicines contained 8 to 56 ingredients. Details regarding the Chinese patent medicines are shown in Table 2. The PCR and sequencing success rate of DG01F/DG01R was 92.86%, as 2 of 26 Chinese patent medicines were not successfully amplified (Fig. 3). Among them, Renshen Zaizao Pill, which is a representative Chinese patent medicine recorded in the Chinese Pharmacopoeia (2015)^1^, shows the most complex ingredients list. In addition to Angelicae sinensis radix, there are 55 other ingredients in this Chinese patent medicine, including Asian Ginseng (Renshen), Pogostemonis Herba (Guanghuoxiang), Santali Albi Lignum (Tanxiang). The primer pair DG01F/DG01R could preferentially amplify the nucleotide signature sequence of Angelicae sinensis radix with clean traces from this Chinese patent medicine. Of the 24 PCR products that were successfully amplified, the nucleotide signature of Angelicae sinensis radix could be detected in 17 batches by direct PCR sequencing, while the other 7 Chinese patent medicines showed messy and overlapping traces.

To determine what was amplified from the other 7 Chinese patent medicines, we conducted TA cloning experiments. Of the 90 total clone sequences, the 37-bp nucleotide signature could be found in 30 sequences. In addition, 48 were found to be unlabelled ingredients in the Chinese patent medicine list. For instance, Notopterygii rhizoma et radix (Qianghuo) was detected in 1 batch of Dingkun Pill and 1 batch of Wuji Baifeng Pill, and *A. gigas* was found in 1 batch of Guipi Pill and 1 batch of Duhuo Jisheng Pill. Other adulterants such as Ligustici rhizoma et radix (Gaoben), Peucedani decursivi radix (Zihuaqianhu) and *A. acutiloba*, were also detected. Another type of adulterant *Carum carvi*, was also found in 2 batches of Niuhuang Qingxin Pills (Table 2). These results indicated that the designed primers can not only amplify Angelicae sinensis radix, but can also amplify the adulterants.

## Discussion

### The necessity of developing the 37-bp nucleotide signature for market supervision

Due to the fact that Chinese patent medicine ingredients are extremely complex, no standardization for Chinese patent medicines has been established. Additionally, because market supervision is not strict and given the lack of regulations in this field, the composition and quality of these medicines and healthcare products are not guaranteed^26^. Moreover, in the Chinese Pharmacopoeia (2015), microscopic and physicochemical identification methods are commonly used, which can detect whether a Chinese patent medicine contains the target species but cannot determine which species contains adulterants. For example, Guipi Pill contains 11 ingredients, 5 of which can be detected by microscopic identification; 3 ingredients are detectable via physicochemical identification using thin layer chromatography. If certain adulterants are present, the identity of the species may not be determinable using current methods.

Molecular identification can be a powerful complement to traditional identification methods in identifying Chinese patent medicines. Yuan *et al*. used it to identify *Angelica* species and demonstrated that the ITS region is the most suitable DNA barcode for identification of this genus, with 100% PCR and sequencing success^27^. They also found that a processing temperature of approximately 80 °C significantly reduced the efficiency of PCR and sequencing. Shaw *et al*.^22^ found that a 121-bp fragment could not be amplified from TCM material after it had been boiled for 120 min. Wuji Baifeng Pill is a Chinese patent medicine worthy of discussion. According to Chinese Pharmacopoeia (2015), the manufacturing process for Angelicae sinensis radix of Wuji Baifeng Pill is as follows: “Put the broken medicines into a jar, add 1500 g of rice wine, seal the jar with a lid and stew the jar in water until no wine is left. Then, pick up the powder and dry it at a low temperature and then crush it into a fine powder”. Using the production process described above, ITS/ITS2 could not be amplified from degraded DNA. Thus, we searched for a nucleotide signature specific to Angelicae sinensis radix within the ITS2 region.

The ideal nucleotide signature should be completely conserved within a specific species. In this region, even the closest related species has one locus that differs from the *A. sinensis* signature. Via Basic Local Alignment Search Tool (BLAST) analysis, we found that the nucleotide signature was not present in any other species. Thus, the nucleotide signature developed in this study can be used as a “species-specific marker” for Angelicae sinensis radix.

### The nucleotide signature effectively identifies Angelicae sinensis radix and its products

As the main adulterant on the market, Angelicae pubescentis radix is another type of Pharmacopoeia-recorded material medica, and its price is lower than that of Angelicae sinensis radix. The two medicinal components share similar morphological characteristics but have different pharmacological properties and medicinal values. Angelicae sinensis radix is mainly used to enrich the blood and promote blood circulation as a dietary therapy, while Angelicae pubescentis radix is known to rheumatism and relieve pain in waist and knee. They should not be used as substitute for the medicine labelled in the patent medicine.

By seeking the nucleotide signature, the percentage of adulterated samples was 26.92% in Chinses patent medicine and 77.78% in “Danggui powder”. In addition to Angelicae sinensis radix and its adulterants, *Carum carvi*, which is not mentioned as a labelled component, was also amplified (169-bp) from 2 batches of Niuhuang Qingxin Pills. This result indicates that *Carum carvi* adulterated with target species Saposhnikoviae radix (Fangfeng) is a very common phenomenon in Chinese patent medicines. In this study, the 167-bp fragment containing the nucleotide signature was successfully amplified from powder, slices, extract powder, decoctions and Chinese patent medicines using the new primer DG01F/DG01R. Samples with the nucleotide signature were successfully identified as Angelicae sinensis radix. This nucleotide signature method is a type of absolute identification, and it will broaden the applications of DNA barcoding for market supervision.

### The prospects for nucleotide signatures using an on-site and real-time detection

Because Angelicae sinensis radix can be identified very accurately and rapidly using the 37-bp nucleotide signature, this method can be developed to provide on-site and real-time detection technology. In recent years, using gold nanoparticles (GNPs) for on-site detection has become a rapidly developing research field. Naoki *et al*. reported a novel method for detecting Hg^2+^ using double-stranded DNA-carrying gold nanoparticles (dsDNA-GNP) within 1 minute with the naked eye^28^. Under the guidance of the basic theory, many scholars have optimized and innovated the above technology, which also inspired us. For instance, Bao *et al*. developed a SNP microarray with capture and detection probes to simply, rapidly, and sensitively assay the genotyping of the samples^29^.

In the future, we can combine GNPs and this 37-bp nucleotide signature method to develop an on-site and real-time detection technology. With this technology, specific detection probes, complementary target sequences and oligonucleotides with this 37-bp nucleotide signature sequence can be designed. Due to the high sensitivity of this technology, its use, with species-specific probes, can be extended to identify degraded DNA samples. Futhermore, we also aimed to develop an inexpensive and user-friendly biological sensor to detect Angelicae sinensis radix with a low limit of detection enabling Chinese patent medicines to be identified in a fast, effective and stable manner.

In conclusion, the nucleotide signature developed in this study provides users with an easy and absolute authentication method and may make a major contribution to the detection of counterfeit products in the Chinese patent medicine market.

## Materials and Methods

### Sample collection and pre-treatment and Data acquisition

A total of 265 samples of *A. sinensis* and its adulterants were collected from China and Japan. The details regarding these samples are shown in Supplementary Table S1. Corresponding voucher samples were deposited in the Herbarium of the Institute of Medicinal Plant Development, Chinese Academy of Medical Sciences, Beijing, China. Fifteen batches of medicinal materials labelled “Danggui slices”, “Danggui powder” and “Danggui extract powder” were purchased from internet stores (Table 1). Twenty-eight batches of Chinese patent medicine containing Angelicae sinensis radix were purchased from stores in Beijing and Guangzhou (Table 2). The ITS2 sequences of *Angelica* genus were downloaded from GenBank to verify their nucleotide signatures. A total of 429 complete ITS2 sequences representing 72 species of *Angelica* were obtained for further study (see Supplementary Table S2).

Decoction: Decoctions were obtained from Angelicae sinensis radix powder. The powdered herb (10 g) was boiled in 300 mL of double-distilled water for 120 min, 180 min and 240 min, and the decoctions were used for DNA extraction.

### DNA extraction, PCR amplification, Sequencing and Cloning

Specimens: The samples were ground into fine powder using a Retsch MM400 laboratory mixer mill (Retsch Co., Germany) at a frequency of 30 Hz. Then genomic DNA was extracted using a Plant Universal Genomic DNA Kit (Tiangen Biotech Beijing Co., China), according to the manufacturer’s instructions. ITS2 was amplified using universal primers^15^.

Decoctions and Chinese patent medicines: Decoctions were centrifuged at 12000× *g* for 3 min to obtain the precipitate for DNA extraction. Chinese patent medicines were subjected to cryogenic grinding in liquid nitrogen using a mortar and pestle. After adding 700 μL of pre-wash buffer (100 mM Tris-HCl, pH 8.0; 20 mM EDTA, pH 8.0; 700 mM NaCl; 2% PVP-40; 0.4% β-mercaptoethanol) to wash the Chinese patent medicine powder several times until the supernatant was clear and colourless, the mixture was centrifuged at 7500× *g* for 5 min at room temperature. Then, genomic DNA was extracted from the precipitate according to the protocol of the Plant Universal Genomic DNA Kit (Tiangen).

A new primer pair, DG01F/DG01R, for amplification of the degraded DNA was designed using Primer Premier 6.0 software (Premier Co., Canada). PCR was performed in a 25-μL reaction system containing 12.5 μL of 2× PCR Master Mix (HT-biotech Co., China), 2.0 μL of each primer (2.5 μM), and 1 μL (about 50 ng) of DNA templates and filled with double-distilled water. The reactions were performed with a thermal cycler (T100^TM^, Bio-Rad, Hercules, CA, USA) using the following thermal program: 94 °C for 5 min, followed by 40 cycles of denaturation (94 °C for 30 s), annealing (53 °C for 30 s), elongation (72 °C for 45 s), and a final 10 min extension at 72 °C. The PCR products were examined using 1.5% agarose gel electrophoresis and purified for bidirectional sequencing using an ABI 3730XL sequencer (Applied Biosystems Co., USA) based on the Sanger sequencing method, at the National Key Facilities for Crop Genetic Resources and Improvement, the Institute of Crop Sciences, Chinese Academy of Agricultural Sciences.

TA cloning experiments: PCR products of Chinese patent medicines were tested via agarose gel electrophoresis, and the desired bands were excised and recovered using a TIANgel Midi Purification Kit (Tiangen). *Taq* DNA polymerase can add an A base at the 3′-end of the PCR products in advance, thus enabling them to be directly ligated to T-vectors. The A-tailed DNA was cloned into *E. coli* DH5α cells (Tiangen) in a pMD^TM^19-T Vector (Takara Bio, Otsu, Japan), according to the manufacturer’s protocol. Ampicillin-resistant single colonies on Luria-Bertani medium were confirmed and selected by PCR with primers DG01F/DG01R. The above cultured clones were used for sequencing at the National Key Facilities for Crop Genetic Resources and Improvement, the Institute of Crop Sciences, Chinese Academy of Agricultural Sciences.

### Sequence analysis

Sequences were edited and assembled manually using CodonCode Aligner 5.1.4 (CodonCode Co., USA). ITS sequences from GenBank were annotated using the Hidden Markov model (HMM) to obtain ITS2 sequences^30^. The haplotypes of *Angelica* species were selected and aligned using MEGA 5.0 software^31^.

## Additional Information

**How to cite this article**: Wang, X. *et al*. A Nucleotide Signature for the Identification of Angelicae Sinensis Radix (Danggui) and Its Products. *Sci. Rep.* **6**, 34940; doi: 10.1038/srep34940 (2016).

## Supplementary Material

Supplementary Information

## Figures and Tables

**Table 1:** Characteristics of the *A. sinensis* samples used in the study.

Sample No.	Latin Name of Original Species	Latin Name of Medicinal Materials	Sample Type	Collection Site	Collection Approach	Identification Result
DGF01	*Angelica sinensis*	Angelicae sinensis radix	Powder	Anhui Bozhou Herb Market	Online	*Angelica biserrata*
DGF03	*Angelica sinensis*	Angelicae sinensis radix	Powder	Anhui Bozhou Herb Market	Online	*Angelica biserrata*
DGF04	*Angelica sinensis*	Angelicae sinensis radix	Powder	Anhui Bozhou Herb Market	Online	*Angelica biserrata*
DGF05	*Angelica sinensis*	Angelicae sinensis radix	Powder	Anhui Bozhou Herb Market	Online	*Angelica biserrata*
DGF06	*Angelica sinensis*	Angelicae sinensis radix	Powder	Anhui Bozhou Herb Market	Online	*Angelica biserrata*
DGF07	*Angelica sinensis*	Angelicae sinensis radix	Powder	Anhui Bozhou Herb Market	Online	*Angelica biserrata*
DGF08	*Angelica sinensis*	Angelicae sinensis radix	Powder	Anhui Bozhou Herb Market	Online	*Angelica biserrata*
DGF02	*Angelica sinensis*	Angelicae sinensis radix	Powder	Anhui Bozhou Herb Market	Online	*Angelica sinensis*
DGF09	*Angelica sinensis*	Angelicae sinensis radix	Powder	Dingxi, Gansu	Online	*Angelica sinensis*
DGT01	*Angelica sinensis*	Angelicae sinensis radix	Extract Powder	Xi’an, Shaanxi	Online	*Angelica sinensis*
DG02	*Angelica sinensis*	Angelicae sinensis radix	Medicinal slices	Dingxi, Gansu	Online	*Angelica sinensis*
DG03	*Angelica sinensis*	Angelicae sinensis radix	Whole Radix	Dingxi, Gansu	Online	*Angelica sinensis*
DG04	*Angelica sinensis*	Angelicae sinensis radix	Whole Radix	Chengdu, Sichuan	Online	*Angelica sinensis*
DG05	*Angelica sinensis*	Angelicae sinensis radix	Medicinal slices	Heze, Shandong	Online	*Angelica sinensis*
DG06	*Angelica sinensis*	Angelicae sinensis radix	Medicinal slices	Dingxi, Gansu	Online	*Angelica sinensis*

**Table 2:** Characteristics of the 28 Chinese patent medicines.

Sample Order	Sample NO.	Sample Name	Collection Site	Amplication Result	Related species in Identification Result
1	ZCY1	Huoxue Zhitong Capsule	Beijing store	Clean traces	*Angelica sinensis*
2	ZCY2	Tianwang Buxin Pill	Beijing store	Clean traces	*Angelica sinensis*
3	ZCY3	Renshen Yangrong Pill	Beijing store	Clean traces	*Angelica sinensis*
4	ZCY4	Renshen Zaizao Pill	Beijing store	Clean traces	*Angelica sinensis*
5	ZCY5	Guipi Pill	Beijing store	Clean traces	*Angelica sinensis*
6	ZCY6	Shenrong Baotai Pill	Beijing store	Clean traces	*Angelica sinensis*
7	ZCY7	Dingkun Pill	Beijing store	Overlapping traces	*Angelica sinensis, Notopterygium incisum*
8	ZCY9	Wuji Baifeng Pill	Beijing store	Overlapping traces	*Angelica sinensis, Notopterygium incisum*
9	ZCY10	Niuhuang Qingxin Pill	Beijing store	Overlapping traces	*Angelica sinensis, Carum carvi*
10	ZCY11	Wuji Baifeng Pill	Beijing store	Clean traces	*Angelica sinensis*
11	ZCY12	Huoxue Zhitong Capsule	Beijing store	Clean traces	*Angelica sinensis*
12	ZCY13	Renshen Jianpi Pill	Beijing store	Clean traces	*Angelica sinensis*
13	ZCY14	Niuhuang Qingxin Pill	Beijing store	Overlapping traces	*Angelica sinensis, Carum carvi*
14	ZCY15	Buzhong Yiqi Pill	Beijing store	None	-
15	ZCY17	Guipi Pill	Guangdong store	Overlapping traces	*Angelica sinensis, Ligusticum sinense, Angelica gigas*
16	ZCY18	Wuji Baifeng Pill	Guangdong store	Overlapping traces	*Angelica sinensis, Ligusticum sinense, Angelica decursiva*
17	ZCY19	Huoxue Tongluo Capsule	Guangdong store	None	-
18	ZCY20	Qixue Shuangbu Pill	Beijing store	Clean traces	*Angelica sinensis*
19	ZCY21	Baihe Gujin Pill	Beijing store	Clean traces	*Angelica sinensis*
20	ZCY22	Duhuo Jisheng Pill	Beijing store	Overlapping traces	*Angelica sinensis, Ligusticum sinense*
21	ZCY23	Pishen Liangzhu Pill	Beijing store	Clean traces	*Angelica sinensis*
22	ZCY24	Qiangli Tianma Duzhong Capsule	Beijing store	Clean traces	*Angelica sinensis*
23	ZCY25	Buzhong Yiqi Pill	Beijing store	Clean traces	*Angelica sinensis*
24	RSJ01	Renshen Jianpi Pill	Beijing store	Clean traces	*Angelica sinensis*
25	RSJ02	Renshen Jianpi Pill	Beijing store	Clean traces	*Angelica sinensis*
26	RSJ03	Renshen Jianpi Pill	Beijing store	Clean traces	*Angelica sinensis*
27	DD01	Dieda Pill	Beijing store	Clean traces	*Angelica sinensis*
28	DD02	Dieda Pill	Beijing store	Clean traces	*Angelica sinensis*
