# Supplementary material for: A Nucleotide Signature for the Identification of Angelicae Sinensis Radix (Danggui) and Its Products
Source: Sci Rep. 2016 Oct 7;6:34940. doi: 10.1038/srep34940 (PMC5054691; doi:10.1038/srep34940)
Supplement: Supplementary Information [file srep34940-s1.pdf]

A Nucleotide Signature for the Identification of Angelicae Sinensis Radix (Danggui) and Its Products

Xiaoyue Wang1, Yang Liu1, Lili Wang1, Jianping Han1*, Shilin Chen2*

1. Institute of Medicinal Plant Development, Chinese Academy of Medicinal Science &Peking Union Medicinal College, Beijing 100193, P.R. China
2. Institute of Chinese Materia Medica, China Academy of Chinese Medical Sciences,
Beijing 100700, P.R. China

Supplementary Table S1 Sampling information of *Angelica sinensis* and its adulterants

Sample No.	Latin Name of Original Species	Latin Name of Medicinal materials	Sample type	Collection Site
DG01	*Angelica sinensis*	Angelicae sinensis radix	Medicinal slices	Beijing City
DG07	*Angelica sinensis*	Angelicae sinensis radix	Whole Radix	Anhui Bozhou Herb Market
DG08	*Angelica sinensis*	Angelicae sinensis radix	Whole Radix	Anhui Bozhou Herb Market
DG09	*Angelica sinensis*	Angelicae sinensis radix	Medicinal slices	Anhui Bozhou Herb Market
DG10	*Angelica sinensis*	Angelicae sinensis radix	Medicinal slices	Anhui Bozhou Herb Market
DG11	*Angelica sinensis*	Angelicae sinensis radix	Medicinal slices	Anhui Bozhou Herb Market
DG12	*Angelica sinensis*	Angelicae sinensis radix	Medicinal slices	Anhui Bozhou Herb Market
DG13	*Angelica sinensis*	Angelicae sinensis radix	Medicinal slices	Anhui Bozhou Herb Market
DG14	*Angelica sinensis*	Angelicae sinensis radix	Medicinal slices	Anhui Bozhou Herb Market
DG15	*Angelica sinensis*	Angelicae sinensis radix	Medicinal slices	Anhui Bozhou Herb Market
DG16	*Angelica sinensis*	Angelicae sinensis radix	Medicinal slices	Anhui Bozhou Herb Market
DG17	*Angelica sinensis*	Angelicae sinensis radix	Medicinal slices	Anhui Bozhou Herb Market
DG18	*Angelica sinensis*	Angelicae sinensis radix	Medicinal slices	Anhui Bozhou Herb Market
DG19	*Angelica sinensis*	Angelicae sinensis radix	Medicinal slices	Anhui Bozhou Herb Market
DG20	*Angelica sinensis*	Angelicae sinensis radix	Medicinal slices	Anhui Bozhou Herb Market
DG21	*Angelica sinensis*	Angelicae sinensis radix	Medicinal slices	Anhui Bozhou Herb Market
DG22	*Angelica sinensis*	Angelicae sinensis radix	Medicinal slices	Anhui Bozhou Herb Market
DG23	*Angelica sinensis*	Angelicae sinensis radix	Medicinal slices	Anhui Bozhou Herb Market
DG24	*Angelica sinensis*	Angelicae sinensis radix	Medicinal slices	Anhui Bozhou Herb Market
DG25	*Angelica sinensis*	Angelicae sinensis radix	Medicinal slices	Anhui Bozhou Herb Market
DG26	*Angelica sinensis*	Angelicae sinensis radix	Medicinal slices	Anhui Bozhou Herb Market
DG27	*Angelica sinensis*	Angelicae sinensis radix	Medicinal slices	Anhui Bozhou Herb Market
DG28	*Angelica sinensis*	Angelicae sinensis radix	Medicinal slices	Anhui Bozhou Herb Market

DG29	*Angelica sinensis*	Angelicae sinensis radix	Medicinal slices	Anhui Bozhou Herb Market
DG30	*Angelica sinensis*	Angelicae sinensis radix	Medicinal slices	Anhui Bozhou Herb Market
DG31	*Angelica sinensis*	Angelicae sinensis radix	Medicinal slices	Anhui Bozhou Herb Market
DG32	*Angelica sinensis*	Angelicae sinensis radix	Medicinal slices	Anhui Bozhou Herb Market
DG33	*Angelica sinensis*	Angelicae sinensis radix	Medicinal slices	Anhui Bozhou Herb Market
DG34	*Angelica sinensis*	Angelicae sinensis radix	Medicinal slices	Anhui Bozhou Herb Market
DG35	*Angelica sinensis*	Angelicae sinensis radix	Medicinal slices	Anhui Bozhou Herb Market
DG36	*Angelica sinensis*	Angelicae sinensis radix	Medicinal slices	Anhui Bozhou Herb Market
DG37	*Angelica sinensis*	Angelicae sinensis radix	Medicinal slices	Anhui Bozhou Herb Market
DG38	*Angelica sinensis*	Angelicae sinensis radix	Medicinal slices	Anhui Bozhou Herb Market
DG39	*Angelica sinensis*	Angelicae sinensis radix	Medicinal slices	Anhui Bozhou Herb Market
DG40	*Angelica sinensis*	Angelicae sinensis radix	Whole Radix	Anhui Bozhou Herb Market
DG47	*Angelica sinensis*	Angelicae sinensis radix	Medicinal slices	Min Country, Gansu Province
DG48	*Angelica sinensis*	Angelicae sinensis radix	Medicinal slices	Beijing City
DG49	*Angelica sinensis*	Angelicae sinensis radix	Medicinal slices	Guangzhou City, Guangdong Province
DGF10	*Angelica sinensis*	Angelicae sinensis radix	Powder	Beijing City
FDC295	*Angelica sinensis*	Angelicae sinensis radix	Powder	National Institutes for food and drug Control
PS1205MT01	*Angelica sinensis*	Angelicae sinensis radix	Original plant	Dingxi Country, Gansu Province
YC0133MT01	*Angelica sinensis*	Angelicae sinensis radix	Medicinal slices	Dingxi Country, Gansu Province
YC0133MT02	*Angelica sinensis*	Angelicae sinensis radix	Medicinal slices	Hebei Anguo Herb Market
YC0133MT03	*Angelica sinensis*	Angelicae sinensis radix	Medicinal slices	Hebei Anguo Herb Market
YC0133MT04	*Angelica sinensis*	Angelicae sinensis radix	Medicinal slices	Hebei Anguo Herb Market
YC0133MT05	*Angelica sinensis*	Angelicae sinensis radix	Medicinal slices	Anhui Bozhou Herb Market
YC0133MT06	*Angelica sinensis*	Angelicae sinensis radix	Medicinal slices	Beijing City
YC0133MT07	*Angelica sinensis*	Angelicae sinensis radix	Medicinal slices	Beijing City
YC0133MT08	*Angelica sinensis*	Angelicae sinensis radix	Medicinal slices	Beijing City
YC0133MT09	*Angelica sinensis*	Angelicae sinensis radix	Medicinal slices	Beijing City
YC0133MT10	*Angelica sinensis*	Angelicae sinensis radix	Medicinal slices	Beijing City
YC0133MT11	*Angelica sinensis*	Angelicae sinensis radix	Medicinal slices	Beijing City
YC0133MT12	*Angelica sinensis*	Angelicae sinensis radix	Medicinal slices	Beijing City
YC0133MT13	*Angelica sinensis*	Angelicae sinensis radix	Medicinal slices	Beijing City
YC0133MT14	*Angelica sinensis*	Angelicae sinensis radix	Medicinal slices	Beijing City
GBDG1	*Angelica sinensis*	Angelicae sinensis radix	Whole Radix	Gansu Province

GBDG2	*Angelica sinensis*	Angelicae sinensis radix	Whole Radix	Gansu Province
GBDG3	*Angelica sinensis*	Angelicae sinensis radix	Whole Radix	Gansu Province
GBDG4	*Angelica sinensis*	Angelicae sinensis radix	Whole Radix	Gansu Province
GBDG5	*Angelica sinensis*	Angelicae sinensis radix	Whole Radix	Gansu Province
GBDG6	*Angelica sinensis*	Angelicae sinensis radix	Whole Radix	Gansu Province
GBDG7	*Angelica sinensis*	Angelicae sinensis radix	Whole Radix	Gansu Province
GBDG8	*Angelica sinensis*	Angelicae sinensis radix	Whole Radix	Gansu Province
GBDG9	*Angelica sinensis*	Angelicae sinensis radix	Whole Radix	Gansu Province
GBDG10	*Angelica sinensis*	Angelicae sinensis radix	Whole Radix	Gansu Province
GBDG11	*Angelica sinensis*	Angelicae sinensis radix	Whole Radix	Gansu Province
GBDG12	*Angelica sinensis*	Angelicae sinensis radix	Whole Radix	Gansu Province
GBDG13	*Angelica sinensis*	Angelicae sinensis radix	Whole Radix	Gansu Province
GBDG14	*Angelica sinensis*	Angelicae sinensis radix	Whole Radix	Gansu Province
GBDG15	*Angelica sinensis*	Angelicae sinensis radix	Whole Radix	Gansu Province
GBDG16	*Angelica sinensis*	Angelicae sinensis radix	Whole Radix	Gansu Province
GBDG17	*Angelica sinensis*	Angelicae sinensis radix	Whole Radix	Gansu Province
GBDG18	*Angelica sinensis*	Angelicae sinensis radix	Whole Radix	Gansu Province
GBDG19	*Angelica sinensis*	Angelicae sinensis radix	Whole Radix	Gansu Province
GBQG1	*Angelica sinensis*	Angelicae sinensis radix	Whole Radix	Yunnan Province
GBQG10	*Angelica sinensis*	Angelicae sinensis radix	Whole Radix	Yunnan Province
GBQG11	*Angelica sinensis*	Angelicae sinensis radix	Whole Radix	Yunnan Province
GBQG12	*Angelica sinensis*	Angelicae sinensis radix	Whole Radix	Yunnan Province
GBQG13	*Angelica sinensis*	Angelicae sinensis radix	Whole Radix	Yunnan Province
GBQG14	*Angelica sinensis*	Angelicae sinensis radix	Whole Radix	Yunnan Province
GBQG15	*Angelica sinensis*	Angelicae sinensis radix	Whole Radix	Yunnan Province
GBQG16	*Angelica sinensis*	Angelicae sinensis radix	Whole Radix	Yunnan Province
GBQG2	*Angelica sinensis*	Angelicae sinensis radix	Whole Radix	Yunnan Province
GBQG3	*Angelica sinensis*	Angelicae sinensis radix	Whole Radix	Yunnan Province
GBQG4	*Angelica sinensis*	Angelicae sinensis radix	Whole Radix	Yunnan Province
GBQG5	*Angelica sinensis*	Angelicae sinensis radix	Whole Radix	Yunnan Province
GBQG6	*Angelica sinensis*	Angelicae sinensis radix	Whole Radix	Yunnan Province
GBQG7	*Angelica sinensis*	Angelicae sinensis radix	Whole Radix	Yunnan Province
GBQG8	*Angelica sinensis*	Angelicae sinensis radix	Whole Radix	Yunnan Province

GBQG9	*Angelica sinensis*	Angelicae sinensis radix	Whole Radix	Yunnan Province
DG41	*Angelica biserrata*	Angelicae pubescentis radix	Medicinal slices	Anhui Bozhou Herb Market
DG42	*Angelica biserrata*	Angelicae pubescentis radix	Medicinal slices	Anhui Bozhou Herb Market
DG43	*Angelica biserrata*	Angelicae pubescentis radix	Medicinal slices	Anhui Bozhou Herb Market
DG44	*Angelica biserrata*	Angelicae pubescentis radix	Medicinal slices	Anhui Bozhou Herb Market
DG45	*Angelica biserrata*	Angelicae pubescentis radix	Medicinal slices	Anhui Bozhou Herb Market
DG46	*Angelica biserrata*	Angelicae pubescentis radix	Medicinal slices	Beijing City
YC0246MT02	*Angelica biserrata*	Angelicae pubescentis radix	Medicinal slices	Hebei Anguo Herb Market
YC0246MT03	*Angelica biserrata*	Angelicae pubescentis radix	Medicinal slices	Hebei Anguo Herb Market
YC0246MT04	*Angelica biserrata*	Angelicae pubescentis radix	Medicinal slices	Hebei Anguo Herb Market
YC0246MT05	*Angelica biserrata*	Angelicae pubescentis radix	Medicinal slices	Beijing City
RC_YC0246MT05	*Angelica biserrata*	Angelicae pubescentis radix	Medicinal slices	Beijing City
YC0246MT06	*Angelica biserrata*	Angelicae pubescentis radix	Medicinal slices	Henan Yuzhou Herb Market
YC0246MT07	*Angelica biserrata*	Angelicae pubescentis radix	Medicinal slices	Northwest Agriculture and Forestry University
YC0246MT08	*Angelica biserrata*	Angelicae pubescentis radix	Medicinal slices	Shaanxi Normal University
YC0246MT09	*Angelica biserrata*	Angelicae pubescentis radix	Original plant	Chongqing City
YC0246MT10	*Angelica biserrata*	Angelicae pubescentis radix	Original plant	Chongqing City
YC0246MT11	*Angelica biserrata*	Angelicae pubescentis radix	Original plant	Chongqing City
YC0246MT12	*Angelica biserrata*	Angelicae pubescentis radix	Original plant	Chongqing City
YC0246MT13	*Angelica biserrata*	Angelicae pubescentis radix	Original plant	Shennongjia City, Hubei Province
FDC078	*Angelica biserrata*	Angelicae pubescentis radix	Powder	National Institutes for food and drug Control
DH	*Angelica biserrata*	Angelicae pubescentis radix	Whole Radix	Hunan Province
YC0152MT03	*Angelica dahurica*	Angelicae Dahuricae Radix	Medicinal slices	Anhui Bozhou Herb Market
YC0152MT05	*Angelica dahurica*	Angelicae Dahuricae Radix	Medicinal slices	Anhui Bozhou Herb Market
YC0152MT06	*Angelica dahurica*	Angelicae Dahuricae Radix	Original plant	Nanchuan District, Chongqing City
YC0152MT07	*Angelica dahurica*	Angelicae Dahuricae Radix	Original plant	Nanchuan District, Chongqing City
YC0152MT08	*Angelica dahurica*	Angelicae Dahuricae Radix	Original plant	Nanchuan District, Chongqing City
YC0152MT09	*Angelica dahurica*	Angelicae Dahuricae Radix	Medicinal slices	Anhui Bozhou Herb Market
YC0152MT14	*Angelica dahurica*	Angelicae Dahuricae Radix	Medicinal slices	Luoyang City, Henan Province
YC0152MT15	*Angelica dahurica*	Angelicae Dahuricae Radix	Original plant	Sipsongpanna, Yunnan Province
RC_YC0152MT03	*Angelica dahurica*	Angelicae Dahuricae Radix	Medicinal slices	Anhui Bozhou Herb Market
RC_YC0152MT14	*Angelica dahurica*	Angelicae Dahuricae Radix	Medicinal slices	Luoyang City, Henan Province
JPHF096	*Angelica dahurica*	Angelicae Dahuricae Radix	Medicinal slices	Tokyo, Japan

FDC003	*Angelica dahurica*	Angelicae Dahuricae Radix	Powder	National Institutes for food and drug Control
RC_YC0106MT02	*Angelica dahurica var. formosana*	Angelicae Dahuricae Radix	Original plant	Nanchuan District, Chongqing City
RC_YC0106MT03	*Angelica dahurica var. formosana*	Angelicae Dahuricae Radix	Original plant	Nanchuan District, Chongqing City
YC0106MT03	*Angelica dahurica var. formosana*	Angelicae Dahuricae Radix	Original plant	Nanchuan District, Chongqing City
YC0106MT04	*Angelica dahurica var. formosana*	Angelicae Dahuricae Radix	Medicinal slices	Guangzhou Qingping Herb Market
YC0106MT05	*Angelica dahurica var. formosana*	Angelicae Dahuricae Radix	Original plant	Nanchuan District, Chongqing City
YC0106MT06	*Angelica dahurica var. formosana*	Angelicae Dahuricae Radix	Original plant	Nanchuan District, Chongqing City
YC0106MT07	*Angelica dahurica var. formosana*	Angelicae Dahuricae Radix	Original plant	Nanchuan District, Chongqing City
YC0106MT08	*Angelica dahurica var. formosana*	Angelicae Dahuricae Radix	Original plant	Nanning City, Guangxi Province
YC0106MT10	*Angelica dahurica var. formosana*	Angelicae Dahuricae Radix	Original plant	Nanning City, Guangxi Province
YC0106MT11	*Angelica dahurica var. formosana*	Angelicae Dahuricae Radix	Original plant	Chengdu City, Sichuan Province
YC0106MT12	*Angelica dahurica var. formosana*	Angelicae Dahuricae Radix	Original plant	Nanning City, Guangxi Province
YC0106MT13	*Angelica dahurica var. formosana*	Angelicae Dahuricae Radix	Medicinal slices	Hangzhou City, Zhejiang Province
ZH1	*Angelica decursiva*	Angelicae Dahuricae Radix	Whole Radix	Hunan Province
ZH2	*Angelica decursiva*	Angelicae Dahuricae Radix	Whole Radix	Hunan Province
YC0449MT02	*Angelica decursiva*	Peucedani Decursivi Radix	Original plant	Nanchuan District, Chongqing City
YC0449MT03	*Angelica decursiva*	Peucedani Decursivi Radix	Medicinal slices	Nanyang City, Henan Province
YC0449MT04	*Angelica decursiva*	Peucedani Decursivi Radix	Original plant	Lushan, Jiangxi Province
YC0449MT05	*Angelica decursiva*	Peucedani Decursivi Radix	Original plant	Lushan, Jiangxi Province
YC0449MT06	*Angelica decursiva*	Peucedani Decursivi Radix	Original plant	Lushan, Jiangxi Province
YC0449MT07	*Angelica decursiva*	Peucedani Decursivi Radix	Medicinal slices	Hebei Anguo Herb Market
YC0449MT08	*Angelica decursiva*	Peucedani Decursivi Radix	Medicinal slices	Hebei Anguo Herb Market
YC0449MT09	*Angelica decursiva*	Peucedani Decursivi Radix	Medicinal slices	Hebei Anguo Herb Market
YC0449MT10	*Angelica decursiva*	Peucedani Decursivi Radix	Medicinal slices	Hebei Anguo Herb Market
YC0449MT11	*Angelica decursiva*	Peucedani Decursivi Radix	Medicinal slices	Hebei Anguo Herb Market
YC0449MT12	*Angelica decursiva*	Peucedani Decursivi Radix	Medicinal slices	Hebei Anguo Herb Market
YC0449MT13	*Angelica decursiva*	Peucedani Decursivi Radix	Medicinal slices	Hebei Anguo Herb Market
YC0449MT14	*Angelica decursiva*	Peucedani Decursivi Radix	Medicinal slices	Hebei Anguo Herb Market
YC0449MT15	*Angelica decursiva*	Peucedani Decursivi Radix	Medicinal slices	Hebei Anguo Herb Market
PS1226MT04	*Angelica decursiva*	Peucedani Decursivi Radix	Original plant	Nanning City, Guangxi Province
PS1226MT05	*Angelica decursiva*	Peucedani Decursivi Radix	Original plant	Nanning City, Guangxi Province
SZ_YC0449MT03	*Angelica decursiva*	Peucedani Decursivi Radix	Medicinal slices	Nanyang City, Henan Province
RC_YC0449MT12	*Angelica decursiva*	Peucedani Decursivi Radix	Medicinal slices	Hebei Anguo Herb Market

GBXJ1	*Conioselinum vaginatum*	–	Whole Radix	Xinjiang Province
GBXJ2	*Conioselinum vaginatum*	–	Whole Radix	Xinjiang Province
GBXJ3	*Conioselinum vaginatum*	–	Whole Radix	Xinjiang Province
GBTDG1	*Levisticum officinale*	Ligustici rhizoma et radix	Whole Radix	Beijing City
GBTDG2	*Levisticum officinale*	Ligustici rhizoma et radix	Whole Radix	Beijing City
GBTDG3	*Levisticum officinale*	Ligustici rhizoma et radix	Whole Radix	Beijing City
GBTDG4	*Levisticum officinale*	Ligustici rhizoma et radix	Whole Radix	Beijing City
GBTDG5	*Levisticum officinale*	Ligustici rhizoma et radix	Whole Radix	Beijing City
YC0482MT01	*Ligusticum jeholense*	Ligustici rhizoma et radix	Powder	Liaoning Institute for Food and Drug Control
YC0482MT02	*Ligusticum jeholense*	Ligustici rhizoma et radix	Medicinal slices	Shenyang City, Liaoning Province
YC0482MT03	*Ligusticum jeholense*	Ligustici rhizoma et radix	Medicinal slices	Shenyang City, Liaoning Province
YC0482MT04	*Ligusticum jeholense*	Ligustici rhizoma et radix	Medicinal slices	Shenyang City, Liaoning Province
YC0482MT05	*Ligusticum jeholense*	Ligustici rhizoma et radix	Medicinal slices	Shenyang City, Liaoning Province
YC0482MT06	*Ligusticum jeholense*	Ligustici rhizoma et radix	Medicinal slices	Shenyang City, Liaoning Province
PS1213MT02	*Ligusticum jeholense*	Ligustici rhizoma et radix	Original plant	Beijing City
CBS508MT01	*Ligusticum jeholense*	Ligustici rhizoma et radix	Original plant	Changbai Mountains
CBS508MT02	*Ligusticum jeholense*	Ligustici rhizoma et radix	Original plant	Changbai Mountains
CBS508MT03	*Ligusticum jeholense*	Ligustici rhizoma et radix	Original plant	Changbai Mountains
FDC378	*Ligusticum jeholense*	Ligustici rhizoma et radix	Powder	National Institutes for food and drug Control
GBLGB1	*Ligusticum jeholense*	Ligustici rhizoma et radix	Whole Radix	Liaoning Proovince
GBLGB2	*Ligusticum jeholense*	Ligustici rhizoma et radix	Whole Radix	Liaoning Proovince
GBLGB3	*Ligusticum jeholense*	Ligustici rhizoma et radix	Whole Radix	Liaoning Proovince
GBLGB5	*Ligusticum jeholense*	Ligustici rhizoma et radix	Whole Radix	Liaoning Proovince
GBLGB6	*Ligusticum jeholense*	Ligustici rhizoma et radix	Whole Radix	Liaoning Proovince
GBLGB7	*Ligusticum jeholense*	Ligustici rhizoma et radix	Whole Radix	Liaoning Proovince
GBLGB8	*Ligusticum jeholense*	Ligustici rhizoma et radix	Whole Radix	Liaoning Proovince
GBLGB9	*Ligusticum jeholense*	Ligustici rhizoma et radix	Whole Radix	Liaoning Proovince
YC0753MT01	*Ligusticum sinense*	Ligustici rhizoma et radix	Medicinal slices	Xi'an City, Shaanxi Province
YC0753MT02	*Ligusticum sinense*	Ligustici rhizoma et radix	Medicinal slices	Xi'an City, Shaanxi Province
YC0753MT03	*Ligusticum sinense*	Ligustici rhizoma et radix	Medicinal slices	Xi'an City, Shaanxi Province
YC0753MT04	*Ligusticum sinense*	Ligustici rhizoma et radix	Medicinal slices	Xi'an City, Shaanxi Province
YC0753MT05	*Ligusticum sinense*	Ligustici rhizoma et radix	Medicinal slices	Xi'an City, Shaanxi Province

YC0753MT06	*Ligusticum sinense*	Ligustici rhizoma et radix	Medicinal slices	Xi'an City, Shaanxi Province
YC0753MT07	*Ligusticum sinense*	Ligustici rhizoma et radix	Medicinal slices	Xi'an City, Shaanxi Province
YC0753MT08	*Ligusticum sinense*	Ligustici rhizoma et radix	Medicinal slices	Xi'an City, Shaanxi Province
YC0753MT09	*Ligusticum sinense*	Ligustici rhizoma et radix	Medicinal slices	Xi'an City, Shaanxi Province
YC0753MT10	*Ligusticum sinense*	Ligustici rhizoma et radix	Medicinal slices	Xi'an City, Shaanxi Province
YC0753MT11	*Ligusticum sinense*	Ligustici rhizoma et radix	Medicinal slices	Xi'an City, Shaanxi Province
YC0753MT12	*Ligusticum sinense*	Ligustici rhizoma et radix	Medicinal slices	Xi'an City, Shaanxi Province
YC0753MT13	*Ligusticum sinense*	Ligustici rhizoma et radix	Medicinal slices	Lanzhou City, Gansu Province
YC0753MT14	*Ligusticum sinense*	Ligustici rhizoma et radix	Medicinal slices	Lanzhou City, Gansu Province
YC0753MT15	*Ligusticum sinense*	Ligustici rhizoma et radix	Medicinal slices	Lanzhou City, Gansu Province
YC0753MT16	*Ligusticum sinense*	Ligustici rhizoma et radix	Medicinal slices	Lanzhou City, Gansu Province
YC0753MT17	*Ligusticum sinense*	Ligustici rhizoma et radix	Medicinal slices	Lanzhou City, Gansu Province
YC0753MT18	*Ligusticum sinense*	Ligustici rhizoma et radix	Medicinal slices	Lanzhou City, Gansu Province
YC0753MT19	*Ligusticum sinense*	Ligustici rhizoma et radix	Medicinal slices	Hebei Anguo Herb Market
FDC377	*Ligusticum sinense*	Ligustici rhizoma et radix	Powder	National Institutes for food and drug Control
PS1208MT01	*Ligusticum sinense*	Ligustici rhizoma et radix	Original plant	Enshi City，Hubei Province
PS1215MT10	*Notopterygium franchetii*	Notopterygii rhizoma et radix	Medicinal slices	Daofu Country, Sichuan Province
PS1215MT11	*Notopterygium franchetii*	Notopterygii rhizoma et radix	Medicinal slices	Xiaojin Country, Sichuan Province
PS1215MT12	*Notopterygium franchetii*	Notopterygii rhizoma et radix	Medicinal slices	Xiaojin Country, Sichuan Province
PS1215MT13	*Notopterygium franchetii*	Notopterygii rhizoma et radix	Medicinal slices	Xiaojin Country, Sichuan Province
PS1215MT14	*Notopterygium franchetii*	Notopterygii rhizoma et radix	Medicinal slices	Dangchang Country, Gansu Province
PS1215MT15	*Notopterygium franchetii*	Notopterygii rhizoma et radix	Medicinal slices	Weiyuan Country, Gansu Province
PS1215MT16	*Notopterygium franchetii*	Notopterygii rhizoma et radix	Medicinal slices	Kangle Country, Gansu Province
PS1215MT02	*Notopterygium franchetii*	Notopterygii rhizoma et radix	Original plant	Kangding Country, Sichuan Province
PS1215MT03	*Notopterygium franchetii*	Notopterygii rhizoma et radix	Original plant	Daofu Country, Sichuan Province
PS1215MT04	*Notopterygium franchetii*	Notopterygii rhizoma et radix	Original plant	Xiaojin Country, Sichuan Province
RC_PS1215MT13	*Notopterygium franchetii*	Notopterygii rhizoma et radix	Medicinal slices	Xiaojin Country, Sichuan Province
RC_PS1215MT14	*Notopterygium franchetii*	Notopterygii rhizoma et radix	Medicinal slices	Dangchang Country, Gansu Province
RC_PS1215MT15	*Notopterygium franchetii*	Notopterygii rhizoma et radix	Medicinal slices	Weiyuan Country, Gansu Province
RC_PS1215MT16	*Notopterygium franchetii*	Notopterygii rhizoma et radix	Medicinal slices	Kangle Country, Gansu Province
FDC287	*Notopterygium franchetii*	Notopterygii rhizoma et radix	Powder	National Institutes for food and drug Control
YC0137MT10	*Notopterygium incisum*	Notopterygii rhizoma et radix	Medicinal slices	China Shineway Pharmaceutical Group Ltd.
YC0137MT11	*Notopterygium incisum*	Notopterygii rhizoma et radix	Medicinal slices	Jilin Lincun Chinese Medicine Development Co., Ltd.

PS2005MT10	*Notopterygium incisum*	Notopterygii rhizoma et radix	Medicinal slices	Rangtang Country, Sichuan Province
PS2005MT11	*Notopterygium incisum*	Notopterygii rhizoma et radix	Medicinal slices	Chengdu Hehuachi Medicine Market, Sichuan Province
PS2005MT12	*Notopterygium incisum*	Notopterygii rhizoma et radix	Medicinal slices	Xiaojin Country, Sichuan Province
PS2005MT13	*Notopterygium incisum*	Notopterygii rhizoma et radix	Medicinal slices	Xiaojin Country, Sichuan Province
PS2005MT14	*Notopterygium incisum*	Notopterygii rhizoma et radix	Medicinal slices	Xiaojin Country, Sichuan Province
PS2005MT15	*Notopterygium incisum*	Notopterygii rhizoma et radix	Medicinal slices	Jinchuan Country, Sichuan Province
PS2005MT16	*Notopterygium incisum*	Notopterygii rhizoma et radix	Medicinal slices	Jinchuan Country, Sichuan Province
PS2005MT17	*Notopterygium incisum*	Notopterygii rhizoma et radix	Medicinal slices	Heishui Country, Sichuan Province
PS2005MT18	*Notopterygium incisum*	Notopterygii rhizoma et radix	Medicinal slices	Kangding Medicine Market
PS2005MT19	*Notopterygium incisum*	Notopterygii rhizoma et radix	Medicinal slices	Kangding Country, Sichuan Province
PS2005MT20	*Notopterygium incisum*	Notopterygii rhizoma et radix	Medicinal slices	Banma Country, Qinghai Province
PS2005MT21	*Notopterygium incisum*	Notopterygii rhizoma et radix	Medicinal slices	Banma Country, Qinghai Province
PS2005MT22	*Notopterygium incisum*	Notopterygii rhizoma et radix	Medicinal slices	Banma Country, Qinghai Province
PS2005MT23	*Notopterygium incisum*	Notopterygii rhizoma et radix	Medicinal slices	Banma Country, Qinghai Province
PS2005MT24	*Notopterygium incisum*	Notopterygii rhizoma et radix	Medicinal slices	Banma Country, Qinghai Province
PS2005MT25	*Notopterygium incisum*	Notopterygii rhizoma et radix	Medicinal slices	Banma Country, Qinghai Province
PS2005MT26	*Notopterygium incisum*	Notopterygii rhizoma et radix	Medicinal slices	Banma Country, Qinghai Province
PS2005MT27	*Notopterygium incisum*	Notopterygii rhizoma et radix	Medicinal slices	Gande Country, Qinghai Province
PS2005MT28	*Notopterygium incisum*	Notopterygii rhizoma et radix	Medicinal slices	Jiuzhi Country, Qinghai Province
PS2005MT29	*Notopterygium incisum*	Notopterygii rhizoma et radix	Medicinal slices	Jiuzhi Country, Qinghai Province
PS2005MT30	*Notopterygium incisum*	Notopterygii rhizoma et radix	Medicinal slices	Menyuan Country, Qinghai Province
PS2005MT31	*Notopterygium incisum*	Notopterygii rhizoma et radix	Medicinal slices	Dari Country, Qinghai Province
PS2005MT32	*Notopterygium incisum*	Notopterygii rhizoma et radix	Medicinal slices	Dawu Country, Qinghai Province
PS2005MT33	*Notopterygium incisum*	Notopterygii rhizoma et radix	Medicinal slices	Dawu Country, Qinghai Province
PS2005MT01	*Notopterygium incisum*	Notopterygii rhizoma et radix	Original plant	Kangding Country, Sichuan Province
PS2005MT02	*Notopterygium incisum*	Notopterygii rhizoma et radix	Original plant	Daofu Country, Sichuan Province
PS2005MT03	*Notopterygium incisum*	Notopterygii rhizoma et radix	Original plant	Kangding Country, Sichuan Province
RC_PS2005MT10	*Notopterygium incisum*	Notopterygii rhizoma et radix	Medicinal slices	Rangtang Country, Sichuan Province
RC_PS2005MT11	*Notopterygium incisum*	Notopterygii rhizoma et radix	Medicinal slices	Chengdu Hehuachi Medicine Market, Sichuan Province
RC_PS2005MT15	*Notopterygium incisum*	Notopterygii rhizoma et radix	Medicinal slices	Jinchuan Country, Sichuan Province
RC_PS2005MT20	*Notopterygium incisum*	Notopterygii rhizoma et radix	Medicinal slices	Banma Country, Qinghai Province
RC_PS2005MT27	*Notopterygium incisum*	Notopterygii rhizoma et radix	Medicinal slices	Gande Country, Qinghai Province
RC_PS2005MT28	*Notopterygium incisum*	Notopterygii rhizoma et radix	Medicinal slices	Jiuzhi Country, Qinghai Province

RC_PS2005MT29	*Notopterygium incisum*	Notopterygii rhizoma et radix	Medicinal slices	Jiuzhi Country, Qinghai Province
RC_PS2005MT30	*Notopterygium incisum*	Notopterygii rhizoma et radix	Medicinal slices	Menyuan Country, Qinghai Province
RC_PS2005MT32	*Notopterygium incisum*	Notopterygii rhizoma et radix	Medicinal slices	Dawu Country, Qinghai Province
FDC286	*Notopterygium incisum*	Notopterygii rhizoma et radix	Powder	National Institutes for food and drug Control
GBXG1	*Seselopsis tianschanica*	−	Whole Radix	Yunnan Province
GBXG2	*Seselopsis tianschanica*	−	Whole Radix	Yunnan Province
GBXG3	*Seselopsis tianschanica*	−	Whole Radix	Yunnan Province
GBXG4	*Seselopsis tianschanica*	−	Whole Radix	Yunnan Province
GBXG5	*Seselopsis tianschanica*	−	Whole Radix	Yunnan Province
GBXG6	*Seselopsis tianschanica*	−	Whole Radix	Yunnan Province
GBXG7	*Seselopsis tianschanica*	−	Whole Radix	Yunnan Province
GBXG8	*Seselopsis tianschanica*	−	Whole Radix	Yunnan Province

Note: The sequences have been submitted to TCM DNA Barcode Database (http://www.tcmbarcode.cn/)

Supplementary Table S2   Accession numbers of GenBank databases

Latin Name of Original Species	GenBank Accession No.
Angelica acutiloba	AB569093 AB697591 AB697592 AB697593 AB697594 AB697595 AB697596 AB697597 AB697598 AB697599 AB697600 AB697601 AB697602 AF169263 AH008024 AJ131291 AY548227 DQ278165 GU395147
Angelica acutiloba var. iwatensis	AB697606 AB697607
Angelica acutiloba var. sugiyamae	AB697603 AB697604 AB697605
Angelica ampla	AH006065 U79598
Angelica amurensis	DQ263581 GU395148
Angelica anomala	DQ263582 GU395149 HQ699461 JX022893
Angelica apaensis	DQ263583 EU001364 EU418373 EU418381 FJ986045 GU395150 HQ686375 HQ686388 JN107553 JX022894 JX022895 JX022896 JX022897 JX022898
Angelica archangelica	AH003539 EF590754 U30576 U30577
Angelica arguta	AH006066 KF619605 U79600
Angelica baizhioides	DQ263588
Angelica balangshanensis	HQ896671
Angelica biserrata	DQ270207 GU395180 JX022899 JX022900 KC812810 KJ999417 KJ999418 KJ999419 KJ999420 KJ999421 KJ999422 KJ999436 KJ999502
Angelica brevicaulis	GU395170
Angelica breweri	U78456
Angelica capitellata	AF009079 KF619735
Angelica cartilaginomarginata	JN603207 JN603208 JN603209 JX022901
Angelica cartilaginomarginata var. distans	AY548222
Angelica cartilaginomarginata var. foliosa	DQ263589 GU395177 JX022906
Angelica cincta	AF009080
Angelica czernaevia	DQ270197 GQ379262 GU395173 JN603210 JN603211
Angelica dabashanensis	HQ896670
Angelica dahurica	AB569095 AF169269 AF169271 AH008027 AH008028 AJ131292 AY925162 DQ278167 EF590755 EU418374 EU591997 FJ980394 GQ434688 GQ434689 GU395151 GU395152 JN603212 JN603213 JN603214 JX022902 JX022903 JX022904 JX022905 JX022907 JX022908 JX022909 JX022940 KP058313 U78476
Angelica dahurica var. formosana	GQ434696 JX022910
Angelica decurrens	AF009078 FJ385033 GU395171

Angelica decursiva	AJ131293 AY548220 DQ132872 DQ263563 DQ263574 DQ263579 EU418375 EU515308 EU592007 EU592012 GQ434705 GQ434706 GU395153 JN603215 JN603216 JN603217 JX022911 JX022912 KF806563 KF806564 KF806565 KF806566 KJ680236 KJ680237 KJ680238 KJ680239 KJ680240 KJ680241 KJ999480 KJ999543 KJ999544 KP334162 KP334163 KP334164 KP334165 KP334166 KP334167 KP334168 KP334169 KP334170 KP334171 KP334172 KP334173 KP334174 KP334175 U78471
Angelica decursiva f. albiflora	HQ256684
Angelica dielsii	GU395154
Angelica duclouxii	GU395155
Angelica fargesii	EU418376 GU395181
Angelica furcijuga	DQ278164 LC035465
Angelica genuflexa	DQ263566
Angelica gigas	AJ131290 DQ263575 DQ263580 GU395156 JN603218 JX022913 KM051435
Angelica grayi	AY146825 AY146891
Angelica hirsutiflora	HQ256683
Angelica japonica	AY548214 DQ278166 JN603219
Angelica kangdingensis	DQ263584 GU395157 JX022914 JX022915 JX022916 JX022917
Angelica keiskei	DQ263561 DQ263562 GU395158
Angelica laxifoliata	DQ263586 EU647210 FJ196889 FJ196890 FJ196891 FJ196892 FJ228464 FJ228466 FJ228468 FJ228469 GU395159 JN107554 JX022918 JX022919 JX022920
Angelica lignescens	AY179030 HQ202011 HQ202012 HQ202013 HQ202014 HQ202015 HQ202016 HQ202017 HQ202018 HQ202019 HQ202020 HQ202021 HQ202022 HQ202023 HQ202024 HQ202025 HQ202026 HQ202027 HQ202028 HQ202029 HQ202030 HQ202031 HQ202032 HQ202033 HQ202034 HQ202035 JF262140
Angelica likiangensis	DQ263587 EU684752 HQ267716 JX022921
Angelica longicaudata	GU395160
Angelica longipes	HQ256679
Angelica lucida	DQ270196
Angelica maowenensis	DQ263585 EU236157 GU395161 JX022922 JX022923 JX022924
Angelica megaphylla	DQ263568 EU418377 GU395162 JX022934 JX022935
Angelica morii	DQ263573 DQ263578 GU395182 JX022925 JX022926
Angelica nitida	DQ263592 EU418378 FJ196887 FJ196888 FJ228465 FJ228467 FJ986041 GU395163 JN107555 JX022927
Angelica omeiensis	DQ263571 DQ263576 GU395164 JX022928
Angelica oncosepala	EU418382

Angelica paeoniifolia	FJ237533 HQ256678
Angelica pinnata	AF358532
Angelica polymorpha	DQ263590 GU395165 GU395179 HQ256680 JN603220 JN603221 JN603222 JN603223 JN603224 JN603225 JX022929 KF806567 U78475
Angelica porphyrocaulis	JX022930
Angelica pseudoselinum	EU236158 EU418379 FJ986042 GU395166 JX022931 JX022932 JX022933
Angelica pubescens	AY925163 DQ263567
Angelica purpurascens	AF009090
Angelica purpureifolia	AY548229
Angelica roseana	AF358533
Angelica sachalinensis	AF077873 DQ263564 U78473
Angelica saxatilis	DQ270195 GU395172
Angelica shikokiana	AB697610 AB697611 HQ256682 LC035466
Angelica sinensis	AF393784 AY140231 AY277247 DQ263570 EU591999 FJ204235 FJ572042 GQ434694 GU289653 GU289654 GU289655 GU289656 GU289657 GU289658 GU395144 JX022936 JX138965 KC295064 KC295071 KF725039 KJ999423 KJ999424 KJ999425 KJ999426 KJ999427 KJ999428 KJ999429 KJ999430 KJ999431 KJ999432 KJ999433 KJ999434 KJ999435 KJ999443 KJ999444 KJ999445 KJ999446 KJ999447 KJ999448 KJ999449 KJ999450 KJ999451 KJ999452 KJ999453 KJ999454 KJ999455 KJ999456 KJ999457 KJ999458 KJ999481 KJ999482 KJ999483 KJ999484 KJ999485 KJ999486 KJ999487 KJ999488 KJ999489 KJ999490 KJ999491 KJ999492 KJ999493 KJ999494 KJ999495 KJ999496 KJ999497 KJ999498 KJ999499 KJ999500 KJ999501 KJ999511 KJ999512 KJ999513 KJ999514 KJ999515 KJ999516 KJ999517 KJ999518 KJ999519 KJ999520 KJ999521 KJ999522 KJ999523 KJ999524 KJ999525 KJ999526 KP334176 KP334177
Angelica songpanensis	GU395167
Angelica sp.	DQ263593 DQ263591 JN603226 JN603227 JN603228 JN603229 JN603230
Angelica stenoloba	AB697608
Angelica stenoloba f. lanceolata	AB697609
Angelica sylvestris	HQ256681 KP682411 U78474
Angelica tatianae	AF009089
Angelica tianmuensis	DQ270194 GU395178 JX022937
Angelica tsinlingensis	DQ263572 DQ263577 GU395168 JX022938
Angelica ursina	DQ263565
Angelica valida	DQ263569 EU418380 GU395169 JX022939
